# Biosynthesis and characterization of silver nanoparticles prepared from two novel natural precursors by facile thermal decomposition methods

**DOI:** 10.1038/srep32539

**Published:** 2016-09-01

**Authors:** Mojgan Goudarzi, Noshin Mir, Mehdi Mousavi-Kamazani, Samira Bagheri, Masoud Salavati-Niasari

**Affiliations:** 1 Young Researchers and Elites Club, Arak Branch, Islamic Azad University, Arak, Iran; 2 Department of Chemistry, University of Zabol, P. O. Box 98615-538, Zabol, Islamic Republic of Iran; 3 Young Researchers and Elites Club, Kashan Branch, Islamic Azad University, Kashan, Iran; 4 Nanotechnology & Catalysis Research Centre (NANOCAT), IPS Building, University of Malaya, 50603 Kuala Lumpur, Malaysia; 5 Institute of Nano Science and Nano Technology, University of Kashan, Kashan, P. O. Box. 87317-51167, I. R. Iran

## Abstract

In this work, two natural sources, including pomegranate peel extract and cochineal dye were employed for the synthesis of silver nanoparticles. The natural silver complex from pomegranate peel extract resulted in nano-sized structures through solution-phase method, but this method was not efficient for cochineal dye-silver precursor and the as-formed products were highly agglomerated. Therefore, an alternative facile solid-state approach was investigated as for both natural precursors and the results showed successful production of well-dispersed nanoparticles with narrow size distribution for cochineal dye-silver precursor. The products were characterized by X-ray diffraction (XRD), Scanning Electron Microscopy (SEM), Energy dispersive X-ray microanalysis (EDX), and Transmission Electron Microscopy (TEM).

As historical anti-infectious agents^1^, silver nanoparticles (SNPs) have always been one of the most intriguing materials for research and developments in order to improve its inherent properties^2^,^3^,^4^,^5^. Coupled with antimicrobial ability of SNPs which plays a vital role in various applications including orthopaedics^6^, packaging^7^, medical devices^8^, footwear, and household items^9^, this nobel nano-metal is well-known for its unique properties which makes it potentially applicable in various fields such as electronic^10^, photonics^11^, photocatalysis^12^, surface-enhanced raman spectroscopy (SERS) detection^13^, biosensor material^14^, etc.

Various methods such as sequential injection^15^, reduction reaction^16^, irradiation-assisted chemical reaction^17^, biosynthesis^18^, and physical methods^19^,^20^, etc have been devised for the synthesis of SNPs with different shapes and sizes. Among all the examined approaches, biosynthesis methods have attracted great interest since it is an environmental friendly and facile approach. The green sources of this technique include a wide range of natural precursors including plant extracts^21^, bacteria^22^, enzymes^23^, and actinomycetes^24^. One of the commonplace sources of antioxidants used for synthesis of silver nanoparticles is fruit juices since they can act both as capping agent and reductant. As one of the rich sources of polyphenols, pomegranate has been a target of many studies for determination of its constituents and attribution of its magical properties to its components available in peel, mesocarp, aril and juice^25^,^26^,^27^. There are certain literature reports on the synthesis of silver nanoparticles from pomegranate juice. Recently, Nadagouda *et al*. reported green synthesis of Ag and Au nanoparticles using antioxidants from blackberry, blueberry, pomegranate, and turmeric extracts. They confirmed formation of more uniform nanoparticles with pomegranate extract in comparison with other studied extracts^28^. Gnana Jobitha *et al*. reported synthesis of SNPs using pomegranate fruit extract as reducing agent^29^. In another recent effort, bimetallic Au/Ag nanoparticles were synthesized at room temperature using the fruit juice of pomegranate and formation of core–shell nanostructures was reported^30^. Synthesis of SNPs from pomegranate peel extract, as a source of polyphenolic substances (condensed tannins, hydrolysable tannins, ellagic and gallic acids, etc.)^31^, has not been reported yet, although there have been some reports on the synthesis of other nanoparticles^32^,^33^.

Another plausible natural source for biosynthesis of nanoparticles, which has been largely neglected so far, is insect natural body product. Carminic acid (CA) is a natural red pigment produced by *Dactylopius coccus* C. insects with a remarkable activity as a radical scavenger comparable to that of known antioxidants as quercetin, ascorbic acid and trolox^34^. Recently, our group^35^ have successfully synthesized CuInS_2_ quantum dot using carminic acid–Cu(II) as a novel copper precursor.

Herein, in continuation of previous works, two different natural sources have been used for the synthesis of silver nanoparticles. First, a solution-phase method is examined and second, a solid-state rout is proposed as an alternative approach for synthesis of monodispresed silver nanoparticles from cochineal-dye precursor.

## Experimental

### Characterization

X-ray diffraction (XRD) patterns were recorded by a Philips-X’PertPro, X-ray diffractometer using Ni-filtered Cu K*α* radiation at scan range of 10 < 2θ < 80. Scanning electron microscopy (SEM) images were obtained on LEO-1455VP equipped with an energy dispersive X-ray spectroscopy. Room temperature photoluminescence^36^ properties were studied on a Perkin-Elmer (LS 55) fluorescence spectrophotometer. The energy dispersive spectrometry^37^ analysis was studied by XL30, Philips microscope. Fourier transform infrared (FT-IR) spectra were recorded on Magna-IR, spectrometer 550 Nicolet with 0.125 cm^−1^ resolution in KBr pellets in the range of 400–4000 cm^−1^. GC-2550TG (Teif Gostar Faraz Company, Iran) were used for all chemical analyses. TEM image was taken with an EM208S Philips transmission electron microscope with an accelerating voltage of 100 kv.

### Synthesis of silver nanoparticles

#### Control sample

All the chemicals used in this method were of analytical grade and used as received without further purification. A control sample consisting of 0.3 g of AgNO_3_ was placed in the furnace, heated up to 300 °C and were kept at this temperature for 3 h. This sample was labelled as Ag-N300.

#### Pomegranate peel extract

The pomegranates (*Punica granatum* L.) used in this experiment were provided from a local garden in Kashan and belongs to the group of pomegranate varieties representing the late season fruit. 1, 2, and 3 grams of dried and powdered pomegranate peel were placed in three beakers and were dissolved in 30 ml water under middle stirring for 30 min and the prepared solutions were filtered off. 0.3 g of AgNO_3_ dissolved in 10 ml water was added to each filtered solution and stirred for 1 min at 80 °C
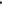
After thermal treatment for 3 h, the system was maintained to cool down to room temperature and the resulting precipitates were collected. Finally, it was washed with absolute ethanol and distilled water for several times and was dried in vacuum at 80 °C for 10 h.

#### Cochineal dye

Cochineal dye was obtained from the dried bodies of female scale insect species, *Dactylopius coccus*, which feed on wild cacti. The insects were dried at 70 °C in an oven until reaching a constant weight. They were finely ground and stored until use. At first, 0.3 g of AgNO_3_ was dissolved in 20 ml of distilled water. Then, 1, 2, and 3 grams of cochineal dye was dissolved in 20 ml of distilled water and filtered through a smooth paper and gradually added into the above solution under magnetic stirring. The mixture was stirred and heated at 80 °C for 1 h. After thermal treatment for 3 h, the system was maintained to cool down to room temperature and the resulting precipitates were collected. Finally, it was washed with absolute ethanol and distilled water for several times and was dried in vacuum at 80 °C for 10 h.

A solid state rout was also investigated for both natural precursors. Different masses of cochineal dye and pomegranate peel were mixed with 0.3 g of AgNO_3_ and the mixtures were well grounded. The resulting powders were placed in a furnace and were heated up to 600 °C for 3 h. The washing step is similar to the already above mentioned methods. Table 1 shows different prepared samples in this work and their experimental conditions.

## Results and Discussion

### Characterization of natural substances

**Figure 1:**
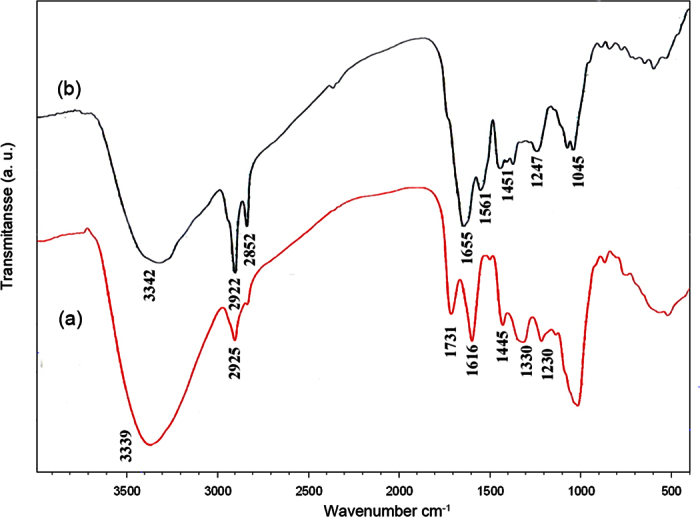
FT-IR spectrum of (**a**) peel powder, (**b**) cochineal dye.

FT-IR spectra of pomegranate peel and cochineal dye are shown in Fig. 1a,b, respectively. In Fig. 1a, the appeared bands are representative of functional groups of various compounds available in pomegranate peel such as phenols, ellagic tannins and gallic and ellagic acid esters^31^. The stretching band at 3394 cm^−1^ corresponds to OH vibration of hydroxyl group. There are two characteristic peaks at 2810 and 2925 cm^−1^ which are attributed to C-H stretching bands. The stretching band at 1731 cm^−1^ could be assigned to C=O stretching of either of lactones, ketones, or carboxylic anhydrides. The C-O stretching vibrations in quinine structure is appeared at 1616 cm^−1^. Other vibrations at 1445, 1333, 1230, and 1030 cm^−1^ are attributed to C=C of aromatic ring, C-H stretching in alkanes or alkyl group, C-O groups stretching in ester, ether, or phenol group, and C-N stretching of aliphatic primary amine, respectively^38^. Figure 1b shows FT-IR spectrum of cochineal dye. The appeared vibrations are in agreement with the previous reports of carminic acid IR spectrum. The broad peak at 3342 cm^−1^ is due to OH stretching vibration and two bands at 2852 and 2922 cm^−1^ are related to C-H stretching vibration of CH_3_ group. The band at 1655 cm^−1^ is assigned to stretching vibration of carminic acid C=O groups. ν_I_(C-C) ν_I/II_(C-C) vibration bands are appeared at 1561 and 1451 cm^−1^, respectively.

### Characterization of as-prepared samples

#### XRD analysis

**Figure 2:**
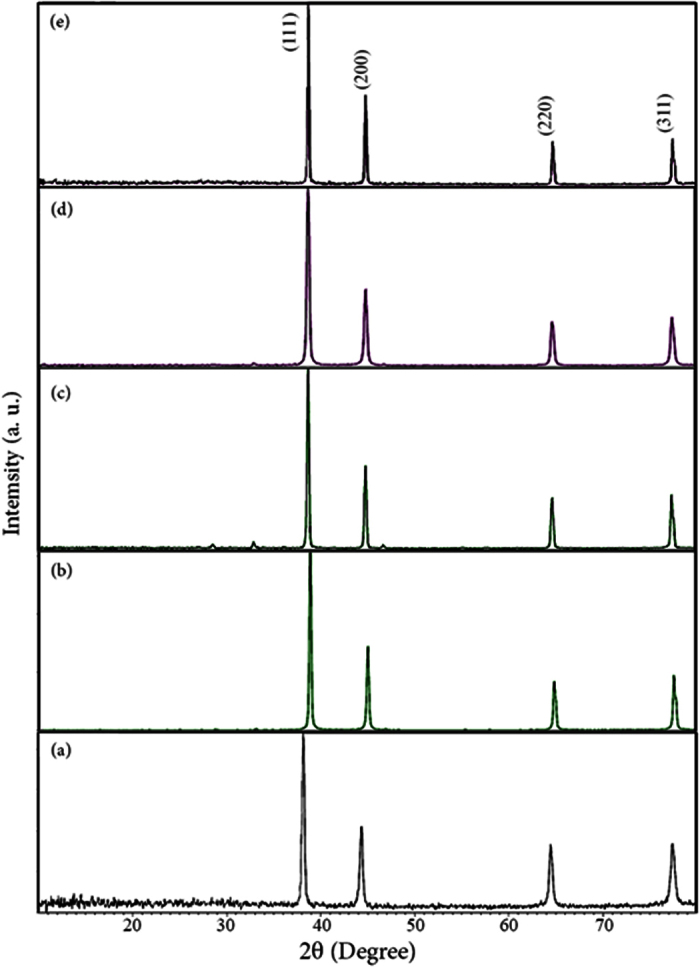
XRD patterns of (**a**) Ag-N300 (**b**) Ag-P300 (**c**) Ag-P600 (**d**) Ag-C300 and (**e**) Ag-C600.

Figure 2a–d show the XRD patterns of Ag nanoparticles obtained from thermal treatment of silver at 300 °C, from solution-phase reaction, and from thermal decomposition of the obtained natural Ag complexes at 300 and 600 °C, respectively. In Fig. 2c,d some weak peaks are observed which could be some organic residual remained from organic or inorganic parts of the precursors^30^,^39^. Apart from that, in all patterns, no other crystalline phases except that silver cubic phase (space group Fm-3m, JCPDS card No. 87–0597) were detected which proves the purity of the products. In Fig. 2a, the XRD pattern of the silver nanoparticles for sample Ag-N300 (the control sample) is clearly presentative of the pure product. From XRD data, the crystallite diameter (D_c_) of Ag nanoparticles were calculated by using the Scherrer equation (D_c_ = Kλ/*β*cosθ), where *β* is the breadth of the observed diffraction line at its half intensity maximum, K is the so-called shape factor, which usually takes a value of about 0.9, and λ is the wavelength of X-ray source used in XRD. The calculated crystalline sizes for all the samples are presented in Table 2. It was observed that Ag-N300 and Ag-P300-2 have the smallest crystalline sizes. As it is well-known, any change in the interlayer of d-spacing of a lattice by organic modification or polymer intercalation results in the shifting of peak position, its broadness, and intensity in the XRD spectra^40^. It seems that increasing the calcination temperature of Ag-P from 300 to 600 has resulted in an increase in crystalline size. Moreover, employing cochineal dye precursor resulted in larger crystalline size.

#### SEM images

**Figure 3:**
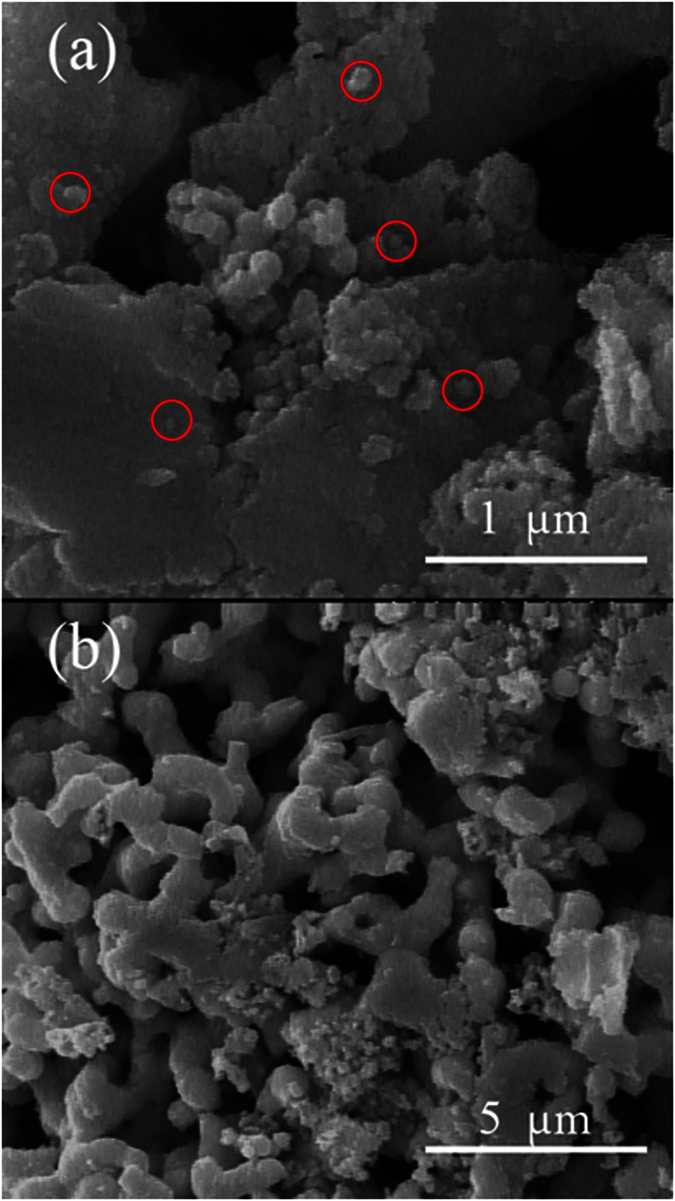
(**a,b**) SEM images of Ag-N300 sample in different magnifications.

In order to investigate the morphology of the as-prepared products, SEM images were recorded for each sample. Figure 3 shows the SEM images of Ag-N300 in two different magnifications. In Fig. 3a small nanoparticles with the size of less than 100 nm are detectable which are shown in red circles. However, from Fig. 3b it is clearly observed that the sample consists of highly agglomerated nanoparticles, which have formed large aggregates with diameter of 400 nm to 2.5 μm.

**Figure 4:**
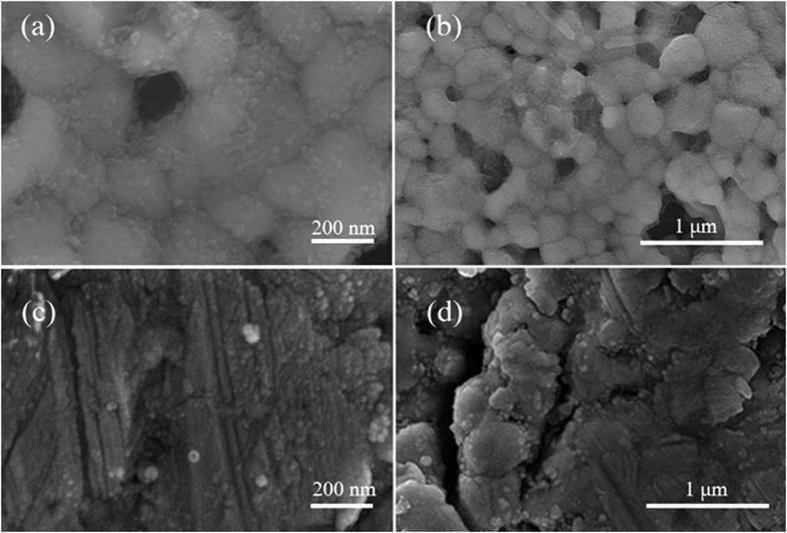
SEM images of (**a**,**b**) Ag-P300, (**c,d**) Ag-P600 samples in different magnifications.

Figure 4a–d show the as-formed samples at different magnifications prepared from silver-pomegranate precursor after calcination at 300 and 600 °C, respectively. Figure 4a,b confirm the formation of the nanoparticles with the size of ca. 140–500 nm. A more detailed look at Fig. 4a reveals a layer of small nanoparticles on the surface of the large ones. In Fig. 4c,d, the Ag-pomegranate precursor have been calcined at 600 °C and it seems that high temperature has caused the nanoparticles to be sintered together and thus, no single detectable nanoparticle can be found. From Fig. 4d, it is clear that the particle size of Ag-P600-1 sample is larger than 1.5 μm.

**Figure 5:**
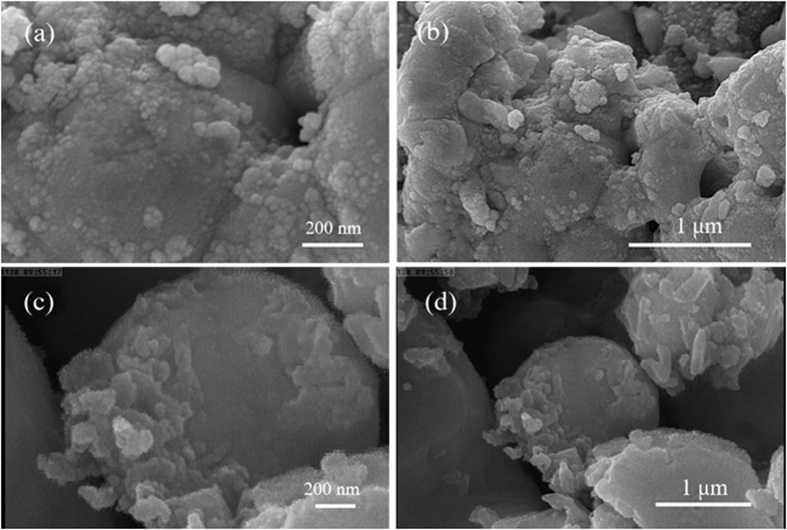
SEM images of (**a**,**b**) Ag-C300, (**c,d**) Ag-C600 samples in different magnifications.

Figure 5a–d show different magnifications of two samples prepared from silver-cochineal dye precursor at 300 and 600 °C, respectively. For Ag-C300-1, Fig. 5a,b show very tiny nanoparticles of Ag-C300-1 sample with the size of 15–40 nm, however, the particles are adhered to each other and have formed very large agglomerations. With increasing the calcination temperature to 600 °C, the morphology has completely changed and no particle has been remained in the sample (Fig. 5c,d). These results suggest that solution-state thermal decomposition method may not be an appropriate route for Ag nanoparticles formation from silver-cochineal dye precursor. Therefore, a solid-state method was examined in order to study the alternative approach for obtaining the desired nanosized product.

#### TEM analysis

**Figure 6:**
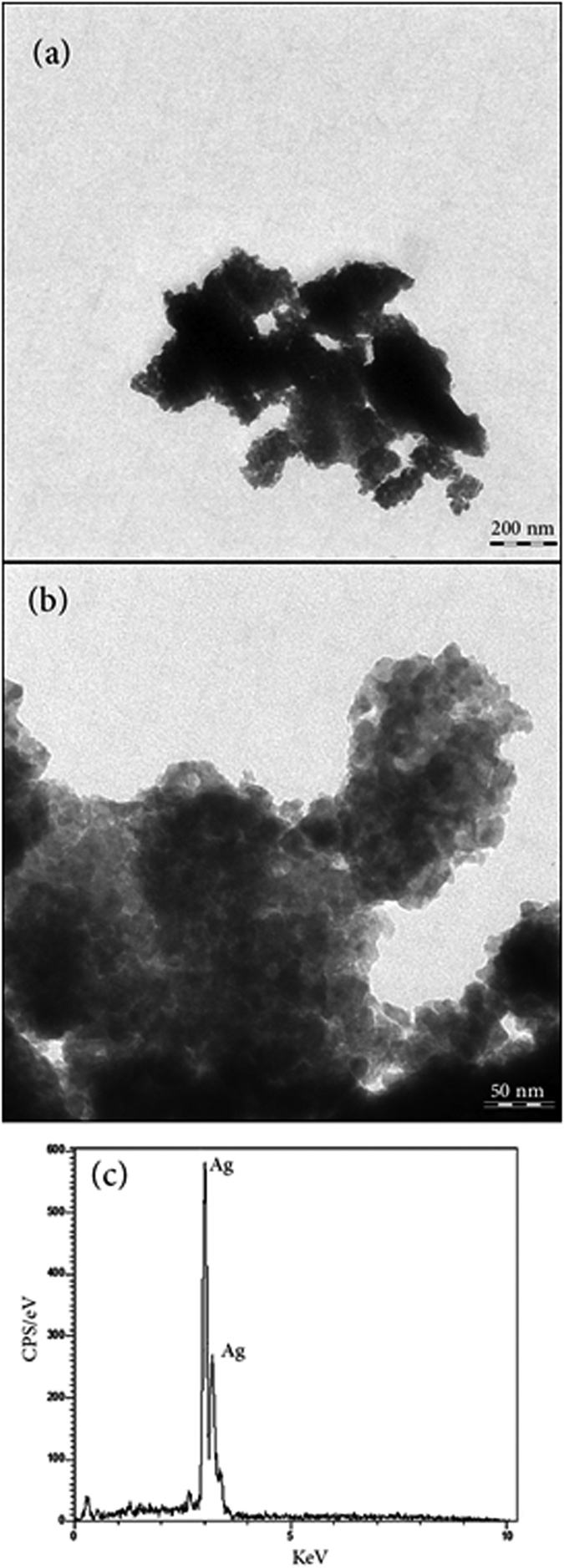
(**a,b**) TEM images of Ag-P300 in two different magnifications, (**c**) EDX of Ag-P300.

In order to better clarify the morphology and size of the particles, TEM analysis was applied. On the basis of SEM images aforementioned above, Ag-P300-2 sample was selected for TEM analysis. Figure 6 shows the TEM images of sample Ag-P300-2 in two different magnifications. The selected section in Fig. 6a which is magnified and is shown in Fig. 6b shows that the sample mainly consist tiny nanoclusters with the size between ca 5–15 nm. The formed nanoclusters are agglomerated and have formed larger particles. It seems that the discussed tiny nanoparticles from SEM image (Fig. 4a,b) are the observed loose nanoclusters here, which are either the broken parts of the large nanoparticles or have not stuck hard enough together to from bigger nanoparticles.

For identification of elemental composition of Ag-P300, EDX analysis was employed and the results were shown in Fig. 6c. It was observed that 100% weight percent of the product is composed of Ag and no other purity exists in the sample.

### Solid state synthesis of Ag nanoparticles by solid silver-pomegranate peel precursor

**Figure 7:**
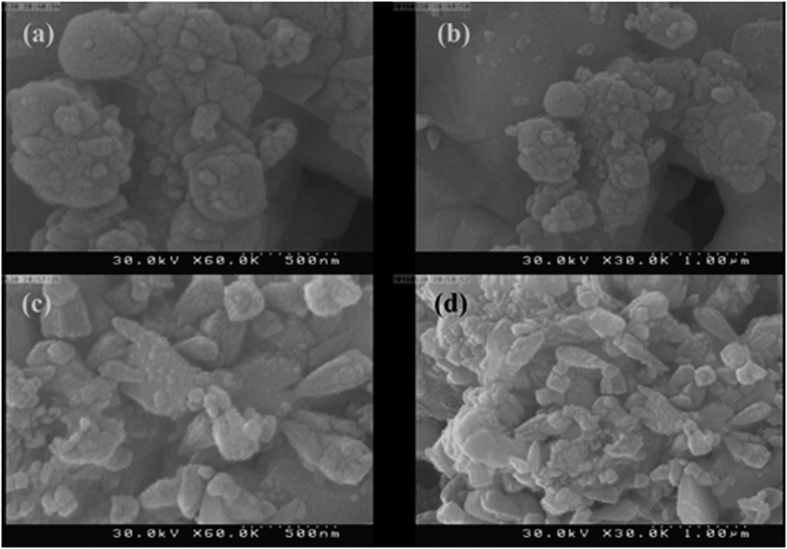
(**a,b**) SEM image of Ag-SP600-1 and Ag-SP600-2 samples, (**c,d**) SEM image of Ag-SP600-1 and Ag-SP600-2 samples.

To investigate alternative approaches for synthesis of Ag nanoparticles from silver-pomegranate peel precursor, two different amounts of pomegranate peel powders were mixed with silver nitrate salt and the ground mixture was calcined at 600 °C for 3 h. Figure 7a–d show two magnifications of the SEM image of Ag-SP600-1 and Ag-SP600-2 samples, respectively. It is observed that both samples are agglomerated at high temperature. However, adding the higher amount of pomegranate peel powder (2 g, Fig. 7c,d) have slightly prevented agglomeration of the particles.

### Solid state synthesis of Ag nanoparticles by solid silver-cochineal precursor

On the basis of the discussion given for SEM images of Ag-C300-1 and Ag-C600-1 (Fig. 5a–d, respectively), it was observed that solution-phase method is not a suitable strategy for obtaining Ag nanoparticles from silver-cochineal dye precursor at neither 300 nor 600 °C Therefore, in order to investigate an alternative approach, this precursor was further prepared in solid phase and was exposed to heat treatment. To ensure complete decomposition of the organic residue, the temperature was set at 600 °C and the as-prepared product (hereafter denoted as Ag-SC600-1) was characterized using various characterization methods.

#### XRD and EDX analyses

**Figure 8:**
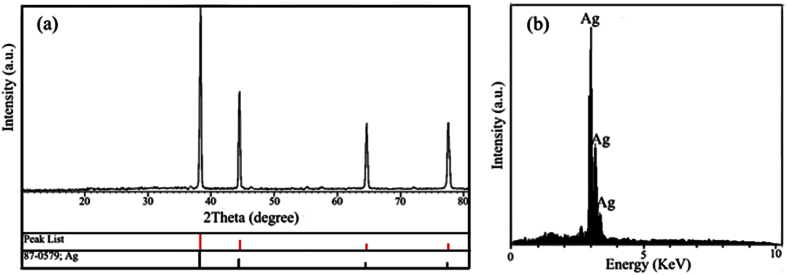
(**a**) XRD pattern and (**b**) EDX analysis of Ag-SC600-1.

Crystalline structure and phase purity of the as-prepared product was determined using XRD and was shown in Fig. 8a. The diffraction peaks observed in Fig. 8a can be indexed to pure cubic phase of silver (a = b = c = 4.0862 Å) with space group of Fm-3 m and JCPDS No. 87-0579. No diffraction peaks from other species could be detected, which indicates the obtained sample is pure. From XRD data, the crystallite diameter (D_c_) of silver nanoparticles was calculated to be 18 nm using the Scherer equation. Chemical purity of product was examined by energy dispersive X-ray spectroscopy (EDX) (Fig. 8b), indicating the existence of pure Ag peaks in the sample.

#### SEM and TEM analyses

**Figure 9:**
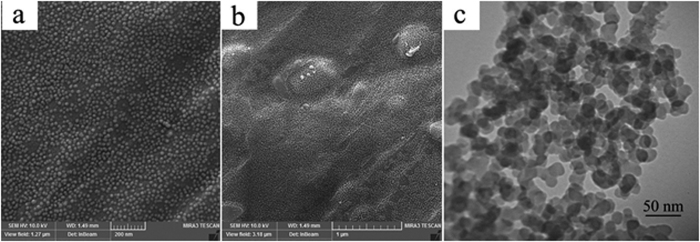
(**a,b**) SEM images and (**c**) TEM image of Ag-SC600-1.

SEM and TEM images were taken for the characterization of the morphology, size, and microstructure of Ag-SC600-1 sample (Fig. 9). Figure 9a,b show very uniform spherical nanoparticles with the average particle size of ca. 15–25 nm which are distinctly separated from each other. TEM image of the sample in Fig. 9c confirms the spherical shape and the uniformity of the nanoparticles. The average particle size from TEM image was estimated to be 20 nm, which is inconsistent with that of observed from SEM images.

#### FT-IR analysis

**Figure 10:**
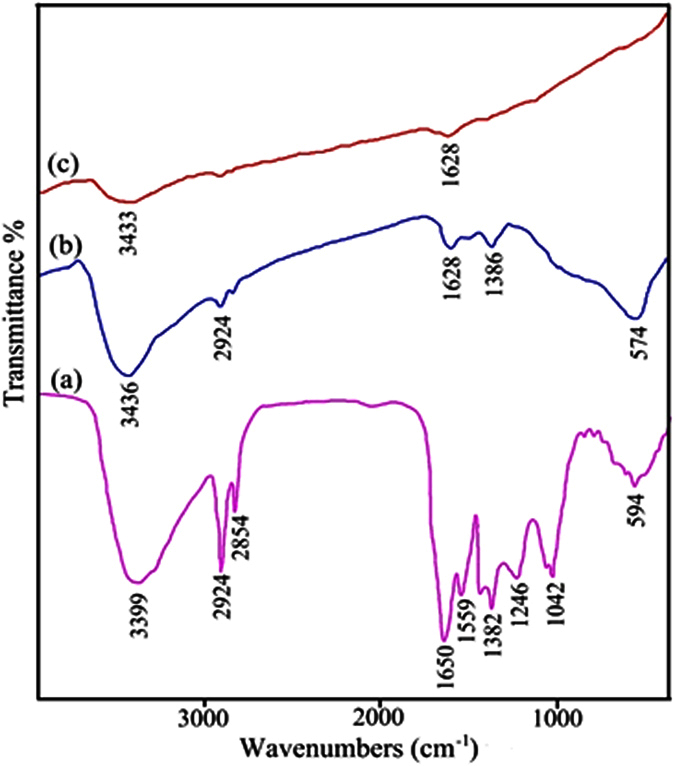
FT-IR spectra of the Ag-SC600-1 sample (**a**) before calcination, (**b**) after calcination, and (**c**) after washing.

Figure 10a–c show the FTIR spectra of the mixture before calcination, after calcination, and after washing with ethanol and water, respectively. As can be seen (Fig. 10a), presence of a significant peak at 3400 cm^−1^ is attributed to the γ(OH) stretching of water available in dye precursor. Moreover, the bands at 2924 and 2854 cm^−1^ are related to C-H stretching and the two other peaks at 1650 cm^−1^ and 1042 cm^−1^ could be attributed to C=O and C-C bonds of the bio-organic compounds available in insect body such as the glucose residue and the carbon chain^41^,^42^,^43^. Furthermore, the absorption peak at 1650 cm^−1^ corresponds to –C=O symmetry stretching vibrations, while the band at 594 cm^−1^ confirms the presence Ag-O bind. The FT-IR spectrum of the calcined sample is illustrated in Fig. 10b. The absorption weak bands at 3436, 2924, 1628, 1386, and 574 cm^−1^ are related to O-H, C-H, C=O, –C=O, and Ag-O bonds, respectively, verifying the presence of organic molecule from cochineal linked to Ag nanoparticles. Therefore, to complete removal of cochineal molecules from the surface of the sample, the sample needs to be washed. According to Fig. 10c, it is obvious that after washing with ethanol and water, there are no important peaks indicating that there is not any organic molecule (from cochineal) absorbed on the surface of the as-synthesized Ag nanoparticles.

### Effect of cochineal dye mass on morphology

**Figure 11:**
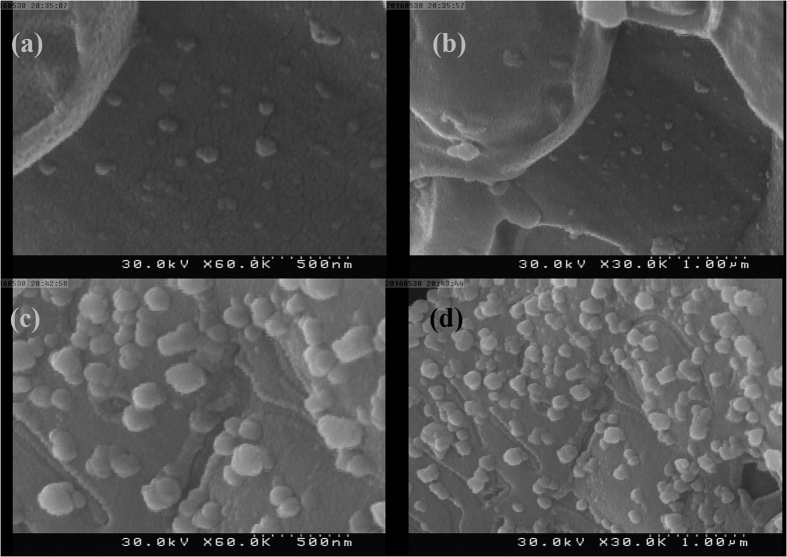
SEM images of (**a,b**) Ag-SP600-1, (**c,d**) Ag-SP600-2 samples in different magnifications.

In order to investigate the effect of cochineal dye amount on the morphology and size of the particles. 2 and 3 g of cochineal dye were also examined. Figure 11a–d show two magnifications of the SEM images of Ag-SC600-2 and Ag-SC600-3, respectively. From the SEM images, it can be clearly observed that with the size of the formed particles has been increased with increasing the amount of cochineal dye. This reveals the key role of the employed natural product on size control of the final product.

### Proposed formation mechanism

**Figure 12:**
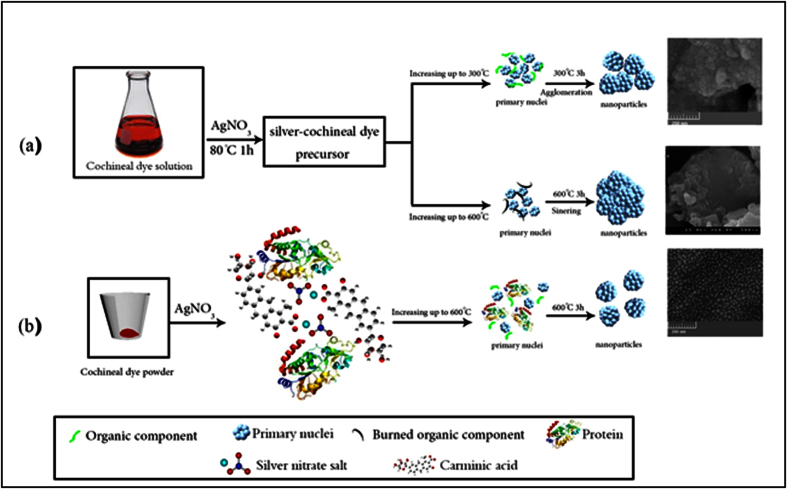
The proposed mechanism for the synthesis of Ag nanoparticles from cochineal dye-precursors via (**a**) solution-phase approach (**b**) solid state approach (The depicted molecules are not in scale).

The principal component of cochineal dye is carminic acid which according to the first studies on this substance, it precipitates nitrate of silver only when it contains nitrogenous substances and results in a reddish precipitate^44^,^45^. Heating the as-formed reddish precipitate at 300 °C, results in formation of small nanoparticles with the size of 15–40 nm. Although the presence of carminate molecules around the silver atoms prevents the initial nuclei from agglomeration, they do not provide enough steric hindrance to prevent the formed nanoparticles from coagulation. Therefore, the result is a very dense collection of small Ag nanoparticles (Fig. 12a). As shown in Fig. 12, calcination of the as-prepared silver-carminate complex in 600 °C did not result in nanosized Ag materials. Due to the high degree of agglomeration, it is likely that after removal of the organic components at ca. 300 °C^46^, the small nanoparticles readily start to sinter together and the microsized spheres are formed. Figure 12b shows the similar procedure in solid phase in a way that the silver nitrate salt was directly mixed with the cochineal powder, was ground in a ceramic mortar and was placed in the furnace at 600 °C for 3 h. The resulting Ag nanoparticles, as shown in Fig. 12b, are well-dispersed spherical ones with narrow size distribution. This significant difference in the resulting products from two methods could be attributed to the different capping agents in the precursors. In the solution-phase approach, silver ions are likely to coordinate to the carminic acid molecules and the rest of the biological products are slightly present in the precursor^47^. However, in the solid-state approach, all of the biological components of the insect including proteins are taking part in the reaction and their high steric hindrance prevents agglomeration of the as-formed nuclei.

**Figure 13:**
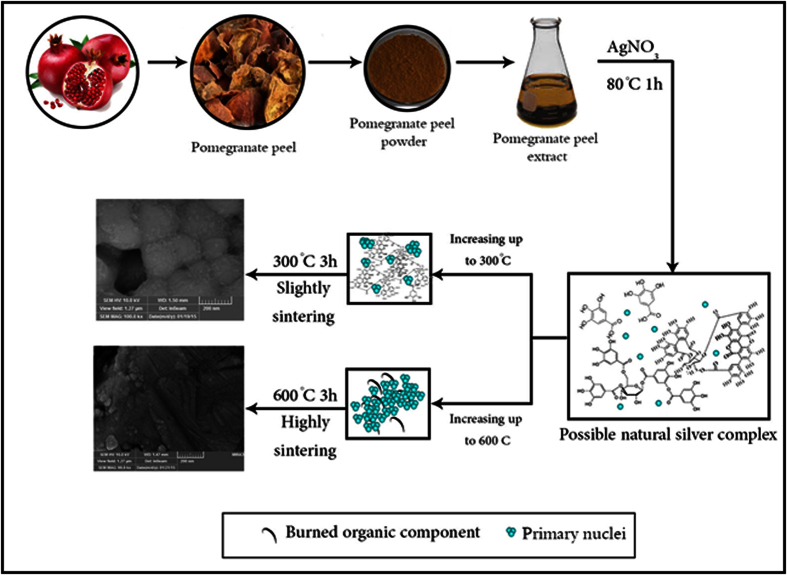
The proposed mechanism for the synthesis of Ag nanoparticles from pomegranate peel-precursors via solution-phase approach approach (The depicted molecules are not in scale).

Pomegranate peel is an important source of polyphenolic substances including condensed tannins, hydrolysable tannins, ellagic and gallic acids, etc^48^. These antioxidant agents are mainly responsible for reduction of the silver salt. Although the most effective phenolic compound in reduction of silver salt has not been determined yet, it is clear that the combination of these agents act as both reductive and capping agents. Although the exact structure of the natural formed complex after the reaction of pomegranate peel extract with silver salt is not determined, it could be the product of coordination of silver ions to the acidic groups of polyphenols, as shown in Fig. 13. Thanks to large bulky chemical structure of the polyphenols available in the pomegranate peel extract including anthocyanin, catechins, punicalin, elagic acid, and punicalagin^48^, the natural silver complex obtained after the reaction, provided enough steric hindrance for the formation of tiny silver nanoclusters. However, burning the organic compounds up to 300 °C results in sintering the as-formed primary nuclei which at 300 °C, particles are slightly sintered while at 600 °C they are strongly sintered and bulk silver is formed (Fig. 13).

Green synthesis of nanoparticles using natural product has been reported in many literatures. Usually the extract of the natural product is used as reducing agent for formation of Ag nanostructure. However to our knowledge, using natural product as a precursor for further synthesis of Ag nanoparticles is not reported. There are also some reports on biosynthesis of silver nanoparticles using bacteria. In all the mentioned methods the variety of morphologies and sizes are formed. Some of the synthesized Ag nanostructures are listed in Table 3. It is observed that in comparison with other methods, solid phase method from silver-cochineal dye precursor results in well-dispersed small nanoparticles.

## Conclusions

In this paper, for the first time, two new biosynthetic sources were employed to obtain Ag nanoparticles. It was shown that in solution phase, pomegranate peel extract form a natural silver complex, which at high temperature (300 °C) transforms to silver nanoparticles. However, the same complex ends up in bulk metallic silver at 600 °C. Moreover, cochineal dye was examined as a natural source for preparation of environmental friendly precursor. It was observed that the obtained precursor from the reaction of aqueous cochineal dye and silver salt does not result in well-dispersed silver nanoparticles. Furthermore, the solid-state reaction of the solid mixture of cochineal dye and silver salt were exposed to high temperature and fascinating, well-dispersed spherical nanoparticles with homogenous size and shape were obtained. This study shows the successful synthesis of the silver nanoparticles with the excellent morphological properties by a very facile, cost effective, and green method.

## Additional Information

**How to cite this article**: Goudarzi, M. *et al*. Biosynthesis and characterization of silver nanoparticles prepared from two novel natural precursors by facile thermal decomposition methods. *Sci. Rep.* **6**, 32539; doi: 10.1038/srep32539 (2016).

## Figures and Tables

**Table 1:** Various prepared samples and their experimental conditions.

Sample	Natural product precursor	Calcination temperature (°C)	Natural product mass (g)	Method
Ag-N300	—	300	—	Solution
Ag-P300-2	Pomegranate peel	300	2	Solution
Ag-P600-2	Pomegranate peel	600	2	Solution
Ag-C300-1	Cochineal dye	300	1	Solution
Ag-C600-1	Cochineal dye	600	1	Solution
Ag-SC600-1	Cochineal dye	600	1	Solid
Ag-SC600-2	Cochineal dye	600	2	Solid
Ag-SC600-3	Cochineal dye	600	3	Solid
Ag-SP600-1	Pomegranate peel	600	1	Solid
Ag-SC600-2	Pomegranate peel	600	2	Solid

**Table 2:** Characteristics of XRD (111) peak for as-prepared samples and the calculated crystalline size from Scherrer equation.

Sample	2θ	FWHM	Crystalline Size
Ag-N300	**38.13**	**0.2952**	**27.8**
Ag-P300	**38.15**	**0.2952**	**27.9**
Ag-P600	**38.13**	**0.2362**	**35.0**
Ag-C300	**38.35**	**0.2362**	**36.0**
Ag-C600	**38.11**	**0.2362**	**35.0**

**Table 3:** Comparison between the results of some silver nanoparticles green synthetic routes with the current study.

No.	Natural source	Morphology	Product size from SEM	Method	Ref.
1	Coffea arabica seed extract a	polymorphic shapes like: rocky, flakes type, spherical, ellipsoidal agglomerates	3–20 μm	solution	49
2	M. paradisiaca peel extract	Irregular shapes	50–150 nm	solution	50
3	potato (Solanum tuberosum) infusion	almost spherical	10–12 nm	solution	51
4	Pomegranate Extract	Aggregated spheres	30–40 nm	solution	29
5	M. dubia leaf extract	irregular shapes	different sizes of nanoparticles	solution	52
6	silver-pomegranate peel precursor	Spherical agglomerated nanoparticles	140–500 nm	solution	This work
7	silver-cochineal dye precursor	Agglomerated nanoparticles	15–40 nm	solution	This work
8	silver-pomegranate peel precursor	Agglomerated nanoparticles	15–50 nm	solid	This work
9	silver-cochineal dye precursor	Uniform spherical nanoparticles	15–25 nm	solid	This work
